# The response of soil eukaryotic microbial communities to afforestation in mountainous area of the Loess Plateau, Northern China

**DOI:** 10.1371/journal.pone.0317235

**Published:** 2025-03-04

**Authors:** Yida An, Lei Zhang, Suqing Li, Yuanyuan Zhang

**Affiliations:** 1 Department of Tourism Management, Xinzhou Normal University, Xinzhou, Shanxi, People’s Republic of China,; 2 Mount Wutai Cultural Research Center, Xinzhou Normal University, Xinzhou, Shanxi, People’s Republic of China,; 3 School of Economics and Management, Taiyuan Normal University, Jinzhong, Shanxi, People’s Republic of China,; 4 Institute of Loess Plateau, Shanxi University, Taiyuan, Shanxi, People’s Republic of China; Jinan University, CHINA

## Abstract

Soil microorganisms are integral to nutrient cycling, ecosystem functioning, and soil restoration. However, the information on the response of soil eukaryotic microbial communities to land-use transformations, particularly for afforestation, remains underexplored in the mountainous region of northwest Shanxi on the Loess Plateau. The study based on high-throughput sequencing of 18S rRNA sequences, elucidated the impact of afforestation on soil eukaryotic microbial communities in this ecologically sensitive region. The findings indicated that afforestation significantly altered the composition of soil eukaryotic microbial communities. The dominant eukaryotic phyla were Streptophyta (16.8%-46.9%) and Ascomycota (20.5%-40.7%). At the genus level, Gymnoascus, Preussia, Mortierella, Chaetomium and Fusarium were biomarkers of soil eukaryotic microbes in farmland soil, while unidentified Streptophyta and Geopora were enriched in plantations soil. The result of non-metric multidimensional scaling (NMDS) analysis shows significant separation between eukaryotic microbial communities in farmland and plantation soils, which significantly correlated with soil temperature (T), nitrate nitrogen (NN) and available phosphorus (AP). These findings provided data support on regional ecological restoration assessments, highlighted the effect of soil physicochemical factors on the composition of soil eukaryotic microbial communities, and enhanced our understanding of the role of afforestation in modifying soil microbial ecosystems.

## 1. Introduction

Soil microorganisms, mainly include bacteria, archaea, fungi, viruses, protozoa and microalgae [1]. Soil microorganisms being abundant and ubiquitous, play a vital role in various aspects of terrestrial ecosystems including soil formation and development, nutrient cycling, plant growth, pollutant degradation, and climate regulation [2]. Moreover, as the key indicator for characterizing soil quality and evaluating soil restoration, soil microorganisms have received widespread attention [3].

Although the importance of soil microorganisms is recognized, the primary emphasis is still typically placed on dominant microbial taxa such as soil bacteria. A lot of work has been carried out on the distribution pattern and driving mechanism of soil bacteria as well as the construction and succession of soil bacterial communities [4,5]. This research provides important theoretical basis to deeply understand the environmental function of soil bacterial communities. However, soil microorganisms include not only dominant bacteria but also other taxa such as soil eukaryotic microorganisms. Unlike the bacteria belonging to a single group, soil eukaryotic microorganisms belong to multiple groups in taxonomy. These organisms mainly include fungi, protozoa, metazoan and microalgae, and assumes a diverse array of roles in trophic food webs and nutrient cycles in soil ecosystem, encompassing primary producers, decomposers, predators and prey [6,7]. To better understand the properties and functions of soil microorganisms, the characteristics related to soil eukaryotic microbial communities have gradually attracted extensive attention [8–10]. Nevertheless, our understanding of the structure and composition of eukaryotic microbial communities is still notably less comprehensive compared to the knowledge of soil bacterial communities.

The severe destruction of natural vegetation and unreasonable land use has led to significant soil erosion, massive loss of soil nutrients, and serious degradation of soil physical properties on the Loess Plateau. The situation in this region has been effectively contained since the implementation of the Grain to Green Program by the Chinese government in 1999. The soil structure and fertility have been enhanced, resulting in a significant improvement in overall soil quality [11]. As an integral component of the soil ecosystem, soil microorganisms inevitably respond to changes in soil quality by adapting to the new environment and performing corresponding functions [12]. Therefore, numerous studies have explored the impact of afforestation on soil bacterial communities and analysed the driving mechanism that controls soil bacterial communities following the afforestation [13,14]. These studies provide a valuable scientific reference for evaluating the effects of ecological restoration in the Loess Plateau. However, in comparison to soil bacteria, detailed information is still lacking on the response of soil eukaryotic microorganisms to the transformation of farmland into plantations on the Loess Plateau, particularly in its northern mountainous regions. Given the widespread presence and crucial ecological functions of soil eukaryotic microorganisms, it is highly significant to explore the diversity and composition features of soil eukaryotic microbial communities in the study area.

The study sampled soil from both farmland and plantations to conduct 18S rRNA high-throughput sequencing, which aimed to: (1) assess the effect of the afforestation on eukaryotic microbial community; (2) determine the influence of soil physiochemical properties on the divergence of eukaryotic microbial communities following afforestation. This study is expected to provide scientific reference for the ecological benefit evaluation of vegetation restoration in the Loess Hilly Region.

## 2. Materials and methods

### 2.1. Sampling area

**Fig 1:**
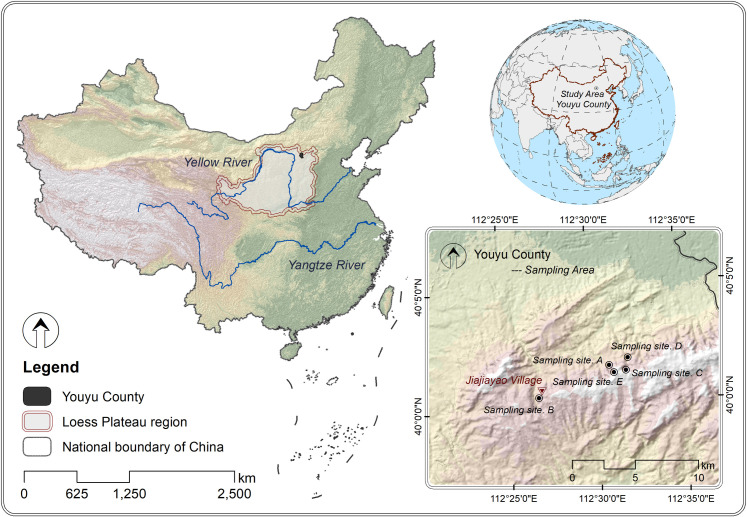
Location map of the sampling site. Sampling site A: farmland planted with Zea mays L.; Sampling site B: farmland planted with Panicum miliaceum L.; Sampling site C: plantation planted with Pinus tabuliformis Carr.; Sampling site D: plantation planted with Populus simonii Carr.; Sampling site E: plantation planted with Caragana korshinskii Kom.

The sampling area (Fig 1) is located in Jiajiayao watershed, Youyu County, Shanxi Province, China (112°27’12.14“E - 112°32’21.74”E, 40°00’29.75”N - 40°01’55.41”N), which is regarded as hilly and mountainous area of the Loess Plateau [15]. The altitude of this region ranges from 1386 m to 1489 m. The average annual temperature is 4.2 °C, and the annual mean precipitation is approximately 410 mm, with most of the rainfall occurring between June and September. The field study was carried out with the permission of local villagers, and did not involve endangered or protected species. The soil type is classified as calcic cambisols according to the FAO-UNESCO soil map of the world [16].

### 2.2. Sampling design

In the sampling area, a large area of farmland has been converted into plantations with the implementation of Grain to Green Program. Therefore, the area of farmland has decreased while that of plantation has gradually expanded. According to the current condition, the two types of sample plots selected in this area are mainly farmland and plantation. The farmland is mainly planted with Zea mays L. (abbreviated as A) and Panicum miliaceum L. (abbreviated as B), and has been cultivated with traditional techniques and low fertilizer input for at least 20 years. The plantation is manually planted with Pinus tabuliformis Carr. (abbreviated as C), Populus simonii Carr. (abbreviated as D), and Caragana korshinskii Kom. (abbreviated as E) for about 15 to 20 years. Neither thinning, pruning nor nutrient amendment was carried out during the growth process of the above plantation.

Three 20 m ×  20 m plots were set in each sampling sites as three biological replicates. The distance between any two plots exceeds 100 m in each sampling site. After removing the litter and humus horizon, a soil auger was used to collect the surface soil (0-10 cm) by using an S-shaped pattern sampling method, with five soil locations collected in each plot. One composite sample per plot was obtained by mixing the five soil samples, and three real replicate samples were obtained in each sampling site. After removing visible stones, animal and plant residues, roots and other substances, all soil samples were sieved through a 2 mm mesh. The sieved soil samples were sealed in sterile plastic bags, placed in an ice-box and transported to the laboratory. All samples above were collected in a sunny day. Finally, the soil sample was divided into two parts: one was used for analyzing soil physiochemical properties, and the other was used for DNA extraction.

### 2.3. Soil physiochemical properties

The method of dichromate oxidation was used to measure soil organic matter (OM), while the Kjeldahl method was employed to measure total nitrogen (TN). Nitrate nitrogen (NN) was measured by ultraviolet spectrophotometer, and ammonium nitrogen (AN) was quantified through the indophenol blue colorimetric method. Alkali-hydrolyzed nitrogen (AHN) was measured by the NaOH-hydrolyzing, NH3-difusing and H3BO3-absorption method. Available phosphorus (AP) was determined by the sodium hydrogen carbonate solution-Mo-Sb anti spectrophotometric method. Available K (AK) was measured using NH4OAc extraction and the flame photometer method. Soil pH (soil-water ratio 1:2) was measured with a pH meter. The soil temperature (T) and moisture (WC) of soil layer at 10 cm were measured using a soil temperature and humidity tester. The detailed analysis procedures were described by Bao [17].

### 2.4. Soil DNA extraction and MiSeq sequencing

Soil genomic DNA was extracted according to the instructions of FastDNA Spin Kit for Soil (MP Biomedicals, USA). DNA quality was evaluated via 1% (w/v) agarose gel electrophoresis and DNA concentration was quantified by NanoDrop spectrophotometer (Thermo Fisher Scientific, USA). The obtained DNA was used to amplify eukaryotic 18S rRNA V4 region via the primer pair 528F and 706R (5’-GCGGTAATTCCAGCTCCAA-3’ and 5’-AATCCRAGAATTTCACCTCT-3’, respectively) [18]. All PCR reaction system was carried out in 30 µ L reactions with 15 µ L of Phusion® High-Fidelity PCR Master Mix (New England Biolabs, USA), 3 µ L of forward and reverse primers (6 µ M), 10 µ L of template DNA and 2 µ L PCR-grade water. Thermal cycling consisted of initial denaturation at 98 °C for 1 min, followed by 30 cycles of denaturation at 98 °C for 10 s, annealing at 50 °C for 30 s, and elongation at 72 °C for 30 s. Finally, 72 °C for 5 min. The amplicons were purified via GeneJET Gel Extraction Kit (Thermo Fisher Scientific, USA). Sequencing libraries were generated using Illumina TruSeq DNA PCR-Free Library Preparation Kit (Illumina, USA) following manufacturer’s recommendations. The library quality was assessed on the Qubit@ 2.0 Fluorometer (Thermo Fisher Scientific, USA) and Agilent Bioanalyzer 2100 system (Agilent Technologies, USA). High-throughput sequencing based on 18S rDNA amplicons was performed on an Illumina NovaSeq platform of Novogene (Beijing, China) and 250 bp paired-end reads were finally generated.

### 2.5. Sequencing data and statistical analysis

Paired-end reads from the original DNA fragments were merged by using FLASH [19]. Paired-end reads were then assigned to each sample according to the unique barcodes. Sequences were subsequently analysed using QIIME (version 1.9.1) [20]. Firstly, reads were filtered by QIIME quality filters, then used pick_de_novo_otus.py to pick operational taxonomic units (OTUs) by making OTU table. Sequences with ≥ 97% similarity were assigned to the same OTUs. The study picked a representative sequence for each OTU and applied the SILVA132 to annotate taxonomic information for each representative sequence.

The bar charts were visualised by using software Origin 2019b. The community composition of eukaryotic microorganisms at the OTU level was analysed by non-metric multidimensional scaling (NMDS) ordinations based on the Bray-Curtis distance using the “vegan” package in R (version 2.15.3). Analysis of similarity (ANOSIM) was conducted to reflect the degree of separation on eukaryotic microbial communities among different sampling plots using the “vegan” package in R. α-diversity was analysed via the “vegan” package in R. Stacked bar chart was conducted via the “ggplot2” package in R according to the relative abundance of eukaryotic microorganisms in each group. Linear discriminant analysis (LDA) effect size (LEfSe) analysis (http://huttenhower.sph.harvard.edu/lefse/) was utilized to find the potential taxonomic biomarkers in each group based on a normalized relative abundance matrix with an LDA score threshold of 4.0. Spearman’s correlation was conducted via the “psych” package in R, the heatmap of Spearman’s correlation analysis was plotted via the “ggplot2” package in R. Redundancy analysis (RDA) was implemented to identify the vital environmental variables influencing the eukaryotic microbial community structure via the “vegan” package, and Mantel test was performed with the “LinkET” package in R. The variation partitioning analysis (VPA) was employed to evaluate the relative contribution of physiochemical parameters on the variance of eukaryotic microbial communities, and performed using the “vegan” package in R. One-way ANOVA was applied to compare the difference of soil physiochemical properties via SPSS (version 19.0).

## 3. Results

### 3.1. Soil physiochemical properties

**Fig 2:**
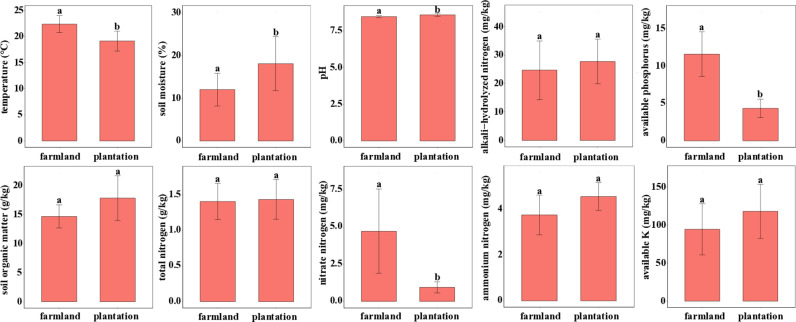
Soil physiochemical properties in farmland and plantation. T-test performed to compare the difference of soil properties between farmland and plantation. Different lowercase letters indicate significant differences at p < 0.05.

The soil physiochemical properties exhibit that there is no significant difference in the content of AHN, OM, TN, AN and AK between the farmland soil and plantation soil (p > 0.05). However, values of other soil physiochemical parameters show significant differences between farmland soil and plantation soil (p < 0.05). In particular, the profile of T, content of AP and NN significantly decreased with the conversion of farmland to plantation (p > 0.05), whereas values of WC and pH significantly increased (p > 0.05). These results suggest that the afforestation led to divergent soil physicochemical conditions (Fig 2).

### 3.2. Soil eukaryotic microbial community analysis

**Fig 3:**
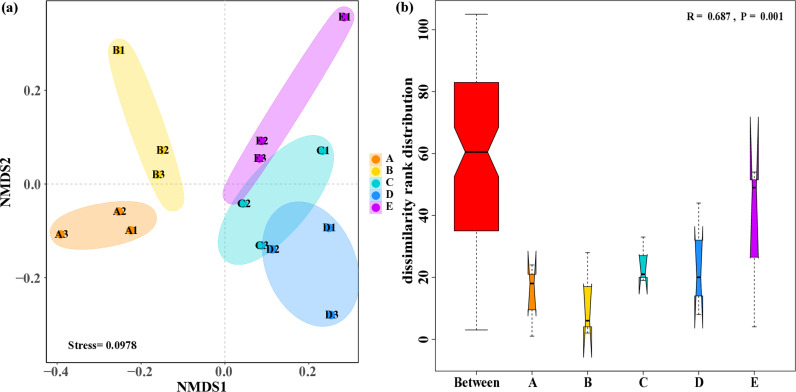
NMDS (a) and ANOSIM (b) analysis: the difference of eukaryotic microbial communities in farmland and plantation. A: farmland planted with Zea mays L.; B: farmland planted with Panicum miliaceum L.; C: plantation planted with Pinus tabuliformis Carr.; D: plantation planted with Populus simonii Carr.; E: plantation planted with Caragana korshinskii Kom.

Alpha diversity and beta diversity can clarify the composition and variation of soil eukaryotic microbial communities in farmland and plantation soil. There is no significant difference on values of diversity index (Simpson and Shannon) for soil eukaryotic microbial communities between farmland and plantation soil (p > 0.05). However, the result of NMDS reflects obvious differences in the composition of eukaryotic microbial communities (Fig 3a). The nearest distance shown between eukaryotic microbial communities from farmland soil planted with A and B, and the cluster is also found among eukaryotic microbial communities from plantation soil planted with C, D, and E. However, the greatest distance lies between the eukaryotic microbial communities in farmland soil and those in plantation soil. The result indicates that soil eukaryotic microorganisms at different sampling sites within farmland or plantation exhibited analogous community structures. A large divergence of soil eukaryotic microbial communities existed between farmland and plantation. The stress value of only 0.0978 signifies that NMDS analysis can accurately represent the degree of difference in soil eukaryotic microbial communities between farmland and plantation. Moreover, the result of the ANOSIM analysis further support the significance of this difference (Fig 3b).

**Fig 4:**
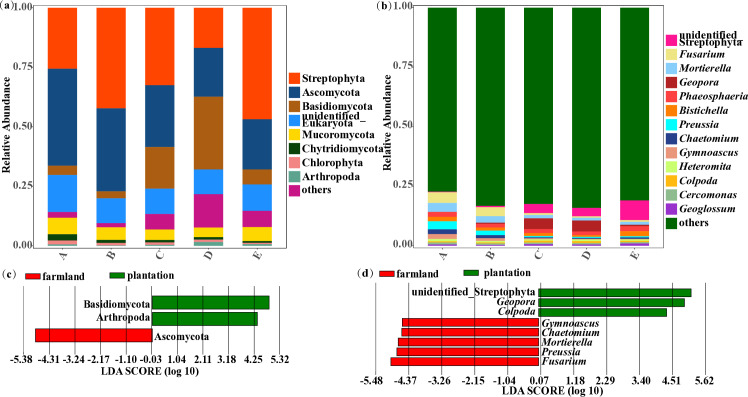
The relative abundance of eukaryotic microbial communities at the level of phylum (a) and genus (b) in farmland and plantation soil. LEfSe analysis of eukaryotic microbial communities with significant differences at the level of phylum (c) and genus (d) in farmland and plantation soil. A: farmland planted with Zea mays L.; B: farmland planted with Panicum miliaceum L.; C: plantation planted with Pinus tabuliformis Carr.; D: plantation planted with Populus simonii Carr.; E: plantation planted with Caragana korshinskii Kom.

A total of 27 eukaryotic phyla were identified in soil samples from farmland and plantation. The dominant eukaryotic phyla (average relative abundance > 0.5%) were Streptophyta (16.8%-46.9%) and Ascomycota (20.5%-40.7%), followed by Basidiomycota (2.93%-30.5%), unidentified_Eukaryota (10.4%-15.6%), Mucoromycota (4.03%-6.92%), Chytridiomycota (0.73%-2.65%), Chlorophyta (0.57%-1.63%) and Arthropoda (0.16%-1.61%) (Fig 4a). The 8 phyla above accounted for 86%-98.2% of eukaryotic microbes in soil samples from farmland and plantation. The average relative abundance of other phyla was relatively low (less than 0.5%). Besides, unidentified_Streptophyta (0.25%-8.26%), Fusarium (0.59%-4.47%), Mortierella (1.16%-3.78%), Geopora (0.018%-4.5%), Phaeosphaeria (1.48%-2.15%), Bistichella (0.73%-2.14%), Preussia (0.43%-3.33%), Chaetomium (0.41%-2%), Gymnoascus (0.16%-2.07%), Heteromita (0.57%-0.97%), Colpoda (0.42%-0.83%), Cercomonas (0.53%-0.8%) and Geoglossum (0.35%-0.94%) predominated and accounted for 15.9%-22.7% of eukaryotic microbes in soil samples from farmland and plantation at the genus level (Fig 4b), and the average relative abundance of other genera accounted for less than 0.5%.

In order to better understand the impact of the afforestation on the composition of soil eukaryotic microbial communities, LEfSe was conducted for taxa with average relative abundance more than 0.5% at the phylum and genus level. Basidiomycota and Arthropoda were enriched in soil from plantation, whereas Ascomycota was enriched in farmland soil (Fig 4c). Fig 4d showed that Gymnoascus, Preussia, Mortierella, Chaetomium and Fusarium were identified as biomarkers of soil eukaryotic microbes in farmland, whereas the biomarkers for soil eukaryotic microbes in plantation were unidentified_Streptophyta, Geopora and Colpoda.

### 3.3. Influences of soil physiochemical properties on eukaryotic microbial communities

**Fig 5:**
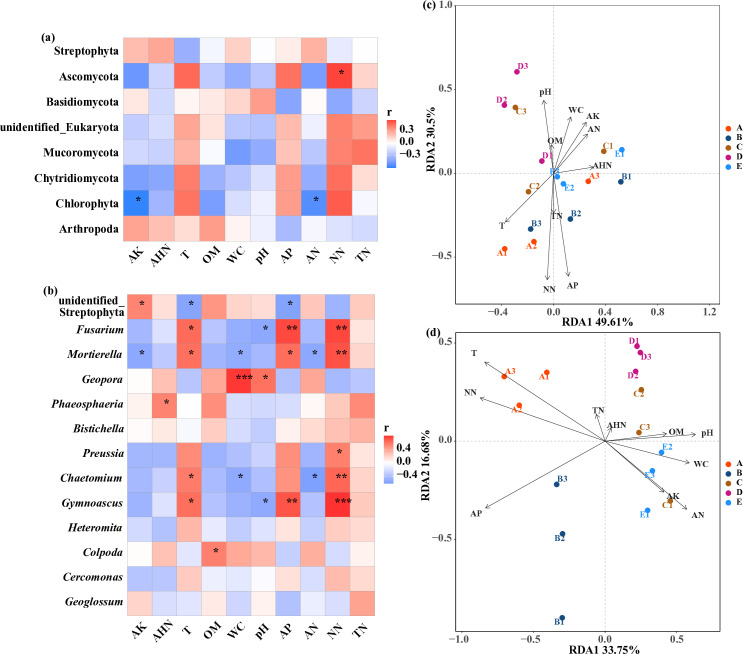
Spearman correlation heatmap about soil physiochemical factors with dominant phyla (a) and genera (b). * p < 0.05, **p < 0.01. RDA of dominant phyla (c) and genera (d) with soil physiochemical factors. T: soil temperature; WC: soil moisture; OM: soil organic matter; NN: nitrate nitrogen; AN: ammonium nitrogen; TN: total nitrogen; AHN: alkali-hydrolyzed nitrogen; AP: available phosphorus; AK: available potassium. A: farmland planted with Zea mays L.; B: farmland planted with Panicum miliaceum L.; C: plantation planted with Pinus tabuliformis Carr.; D: plantation planted with Populus simonii Carr.; E: plantation planted with Caragana korshinskii Kom.

Heatmap of Spearman correlation is performed to reveal the relationship between soil eukaryotic microbial communities and soil physiochemical properties. Fig 5a denotes that only the abundance of Ascomycota and Chlorophyta affected by soil physiochemical properties. There is a significant positive correlation between the abundance of Ascomycota and NN (p < 0.05), and the abundance of Chlorophyta is significantly negatively correlated with AN and AK (p < 0.05). Meanwhile, the abundance of most genera (average relative abundance > 0.5%) is significantly correlated with soil physiochemical properties except for Bistichella, Heteromita, Cercomonas and Geoglossum (Fig 5b). Furthermore, T, NN and AP are closely related to soil eukaryotic microbial communities, these factors could affect 6 of the 9 genera that significantly responded to changes in soil physiochemical properties.

**Fig 6:**
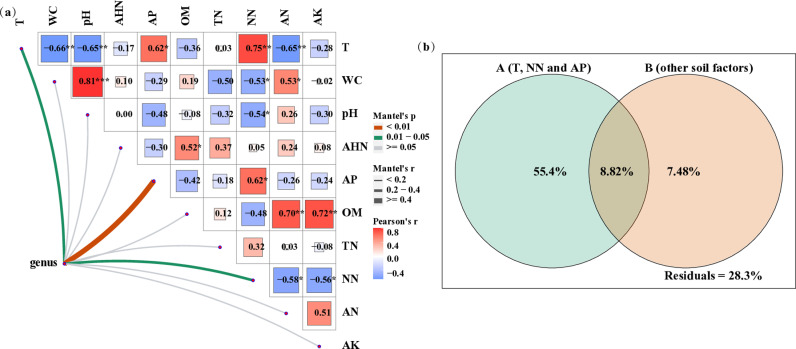
Mantel test (a) of soil eukaryotic microbial communities and physiochemical parameters. *p < 0.05, **p < 0.01. VPA (b) indicate the relative contributions to variation in soil eukaryotic microbial communities explained by soil physiochemical factors. T: soil temperature; WC: soil moisture; OM: soil organic matter; NN: nitrate nitrogen; AN: ammonium nitrogen; TN: total nitrogen; AHN: alkali-hydrolyzed nitrogen; AP: available phosphorus; AK: available potassium.

RDA method was applied to evaluate the response of soil eukaryotic microbial communities to soil physiochemical properties. As depicted in Fig 5c, RDA1 and RDA2 respectively accounted for 49.61% and 30.5% of the total eukaryotic microbial variation at the phylum level. The first and second axes accounted for 50.43% of the variation at the genus level (Fig 5d). The result indicates that soil physiochemical properties have great influences on soil eukaryotic microbial communities. Moreover, eukaryotic microbial communities in farmland soil had higher correlations with T, TN, NN and AP, whereas eukaryotic microbial communities in plantations soil showed higher correlations with OM, AN, AHN, AK, pH and WC. Out of the chosen variables, T, NN, and AP are the key factors that showed significant correlations (p < 0.05) with eukaryotic microbial communities (Fig 6a), which suggests that their variation can significantly affect soil eukaryotic microbial communities. Moreover, the VPA result reveals that soil physiochemical properties could explain 71.7% of the total variation of soil eukaryotic microbial communities (Fig 6b). Among them, soil physiochemical properties of T, NN and AP could explain 64.2% of the total variation while the other could explain 16.3%, whereas the overlap could only explain 8.82% of the total variation. It is apparent that the influence of soil physiochemical properties of T, NN and AP on soil eukaryotic microbial communities outweigh soil physiochemical properties of others.

## 4. Discussion

### 4.1. The response of soil physiochemical properties to the afforestation

The afforestation is a critical and effective avenue to recover degraded ecosystems in the Loess Plateau of northern China [21]. The process has been also reported to affect soil physiochemical properties [22]. In line with existing research, this paper also discovered the response of soil physicochemical properties to afforestation. After the afforestation, T, content of AP and NN significantly decreased, while WC and pH significantly increased. For this finding, with the change of vegetation type, the primary production, carbon input in the form of litter and root exudates, as well as moisture and temperature will inevitably been influenced [23,24]. Moreover, owing to the absence of cultivation, the level of disturbance to the plantation soil remains relatively low, thereby influencing its biogeochemical cycling processes [25]. Therefore, the change of soil environment as described above is likely to be the vital consideration leading to differential responses of soil physiochemical properties to the afforestation.

### 4.2. Effect of the afforestation on soil eukaryotic microbial communities

At the phylum level, Streptophyta, Ascomycota, and Basidiomycota were the predominant eukaryotic taxa in both farmland and plantation soil, corroborating previous reports on soil eukaryotic microbial communities in the Loess Plateau [26,27]. Streptophyta, often regarded as a proxy for active photosynthetic microorganisms, and might contribute to accumulating soil nutrients [28]. Ascomycota and Basidiomycota, being predominantly saprophytic, serve as primary decomposers of plant cellulose, lignin, and other recalcitrant organic matter [26]. Consequently, they occupy a pivotal position in the soil nutrient cycle. Additionally, Basidiomycota, as integral members of mycorrhizal fungi, have the ability to counteract the adverse effects of root pathogens on plant growth, thereby promoting a healthy soil microbial ecological environment [29]. Similarly, Mucoromycota, Chytridiomycota and Chlorophyta affiliating to fungi and microalgae are also beneficial to soil nutrients accumulation and soil nutrients cycle [30,31]. Arthropoda belonging to the metazoans, which is the dominant phyla across all soil samples in the Loess Plateau [6], which is proved again in this study. Nematoda typically predominates in soil and contributes positively to ecological processes including nitrogen cycling, decomposition, and disease suppression [6,32]. However, its abundance is relatively low (<0.5%) in both farmland and plantation soil, which might be associated with the relatively poor soil nutrition in the study area. Moreover, the relative abundance of Basidiomycota and Arthropoda significantly increased after the afforestation. Previous studies have indicated that Basidiomycota rely on decaying wood components to obtain sufficient carbon sources for expand living space or form mycorrhizae [33]. Therefore, it is speculated that the stable environment of plantation soil and the accumulation of plant residues promote the growth of Basidiomycota. However, tillage practices have been found to limit the abundance of soil microarthropods [34], which might be a considerable reason contributing to the low abundance of Arthropoda in farmland soil.

At the genus level, the majority of dominant genera belong to fungi, which play a crucial role in soil health and nutrient cycling [35]. Certain protozoan taxa also held dominance, exerting control over diverse soil microorganisms and nutrient cycling [36]. Gymnoascus, Preussia, Mortierella, Chaetomium, and Fusarium were enriched in farmland soil, whereas Geopora and Colpoda were enriched in plantation soil. Fusarium is a pathogenic microorganism that can multiply rapidly in soil, and root rot caused by it is a significant factor in reducing crop and tree yields [37]. Apparently, the afforestation can significantly decrease the relative abundance of Fusarium in the soil, which aiding in the reduction of the likelihood of plant diseases. Mortierella is a prominent group of eukaryotes in farmland soil, which is consistent with the results of existing research [38]. This species of fungi possesses a robust ability to decompose cellulose, thrives in relatively barren soils, and has positive effects on improving soil nutrient availability and structure [39]. The abundance of Mortierella is relatively higher in farmland soil compared to plantation soil due to its properties. However, some species of Mortierella are plant pathogenic fungi that can cause the death of plant seedlings [40]. This suggests the risk of fungal diseases in farmland, thus necessitating more precautions during the cultivation process. Chaetomium commonly assumes a significant role in the degradation of plant cellulose [41]. The enrichment of the taxon could potentially facilitate the provision of organic matter in farmland soil. Similarly, Geopora belong to saprophytic fungi that participate in soil nutrition cycle [42]. Consequently, the enrichment of Geopora in plantation soil is beneficial to plant growth. Soil ciliate can be used as an ideal index to evaluate the ecological restoration effect [43]. As one of the groups, Colpoda probably tended to be enriched in plantation soil, which had abundant nutrients.

### 4.3. The relationship between soil eukaryotic microbial communities and soil physiochemical properties

Numerous studies have demonstrated that soil physiochemical properties have significant impacts on microbial communities [44,45]. The results of this paper indicate that the content of AP is one of key factors leading to the difference of eukaryotic microbial communities, which is consistent with the existing studies [46,47]. In addition, the decrease in AP content during the restoration process can be attributed to soil biological phosphorus metabolism from both plants and microbes. [47]. On the other hand, soluble phosphate ions can combine with positively charged metal ions in alkaline soil, which are adsorbed on the surface of soil particles and in turn restrict the mobility and availability of phosphorus in soil [48]. In the research, NN is also one of the vital factors leading to the divergence of eukaryotic microbial communities. Furthermore, afforestation has led to an increase in the abundance of denitrifying functional genes and a decrease in the content of NN [49]. Fungi are more sensitive to nitrogen additions than bacteria [50]. Moreover, the fungi is primarily responsible for decomposing complex compounds with higher C/N ratios, and making them better suited for habitats with higher C/N ratios and nitrogen deficiencies [51]. Therefore, it is speculated that the response of eukaryotic microbial communities to NN is likely derived from the denitrification process induced by soil bacteria. Besides, the impact of NN on eukaryotic microbial communities mainly achieved by regulating fungal groups taxa (Fig 5a and Fig 5b). According to the existing literature, variations in soil eukaryotic microbial communities were significantly related to soil temperature [52], while the development of vegetation resulting from reforestation efforts has been shown to lower soil temperature [53], and Fungi are better adapted to low temperature conditions than bacteria [54]. Consequently, T is considered to be a crucial factor that significantly affecting soil eukaryotic microbial community.

On the other hand, OM content, WC, AK and pH have also been proved to be key factors affecting the composition of soil eukaryotic microbial communities [55,56]. Although these properties did not significantly affect soil eukaryotic microorganisms at the community level in this research, but they still exhibited significant positive or negative correlations with certain genera. As a result, these soil parameters can be regarded as secondary influencing factors of soil eukaryotic microbial communities in the study area.

### 4.4. Implications of afforestation on soil health

Many studies have demonstrated that the composition of soil microorganisms is crucial for maintaining soil health and ecosystem functioning [57,58]. In this research, both Basidiomycota and Arthropoda were notably enriched in plantation soils. The increased presence of these taxa not only promotes soil nutrient cycling, but also indicates a favorable soil quality status. In contrast to these beneficial microorganisms, plant pathogenic fungi such as Fusarium and Mortierella were significantly suppressed in plantation soils. These responses suggest that prolonged and consistent afforestation has effectively leveraged microorganisms to optimize soil internal functions, thereby playing a critical part in maintaining soil health and supporting the sustainable development of soil ecosystems.

## 5. Conclusion

Based on the detection of soil eukaryotic microbial communities via 18S rRNA high-throughput sequencing, the study demonstrated that afforestation significantly reshaped soil eukaryotic microbial communities of sampling sites at the phylum and genus level, with significant differences observed between farmland and plantation soils. Moreover, T, NN, and AP emerged as key factors significantly affecting the composition of eukaryotic microbial communities. These insights are instrumental for advancing regional ecological restoration strategies, and helping us to understand afforestation’s role in transforming soil microbial ecosystems in the Loess Plateau.

## Supporting information

S1 File R code used in this paper. (DOCX)
