# Supplementary material for: The response of soil eukaryotic microbial communities to afforestation in mountainous area of the Loess Plateau, Northern China
Source: PLoS One. 2025 Mar 4;20(3):e0317235. doi: 10.1371/journal.pone.0317235 (PMC11878901; doi:10.1371/journal.pone.0317235)
Supplement: S1 File — (DOCX) [file pone.0317235.s001.docx]

**R code used in this paper**

1. **# Undisplayed Alpha Diversity**

library(vegan)

OTU = read.delim('Tax.txt', row.names = 1, sep = '\t', stringsAsFactors = FALSE, check.names = FALSE)

shannon = diversity(OTU,MARGIN = 1)

simpson = diversity(OTU,index = "simpson",MARGIN = 1)

invsimpson = diversity(OTU,index = "invsimpson",MARGIN = 1)

SR = specnumber(OTU,MARGIN = 1)

Pielou = shannon/log(SR)

diversity = data.frame(shannon,simpson,SR,Pielou,invsimpson)write.csv(diversity,"diversity.csv")

library(ggplot2)

OTU = rrarefy(OTU,min(rowSums(OTU)))

Shannon.wiener = diversity(OTU, "shannon")

Simpson = diversity(OTU,"simpson")

Inverse.Simpson = diversity(OTU, index = "inv")

S = specnumber(OTU)

J = Shannon.wiener/ log(S)

ca = data.frame(estimateR(OTU))

X = data.frame(J,S,Inverse.Simpson,Simpson,Shannon.wiener)

X

class(Shannon.wiener)

Shannon.wiener = as.data.frame(Shannon.wiener)

p = ggplot(Shannon.wiener, aes(x=groups, y=shannon),color=groups) +

geom_boxplot(aes(fill=factor(groups))) +

theme(axis.text.x=element_text(angle=50,hjust=0.5, vjust=0.5))

1. **Figure 3.** NMDS (a) and ANOSIM (b) analysis illustrating the difference of eukaryotic microbial communities in farmland and plantation.

#Figure 3 (a) NMDS

#Load R package

library(openxlsx)

library(vegan)

library(ggplot2)

library(ggrepel)

#Read data

spe = read.xlsx('OTU.xlsx', rowNames = T)

# Perform a Hellinger transformation before sorting multidimensional data

spe = decostand(spe, method = 'hellinger')

#Calculate bray_Curtis distance

otu.distance = vegdist(spe, method = 'bray')

#NMDS sorting analysis used the metaMDS function in the vegan package

df_nmds = metaMDS(otu.distance, k = 2)

#Stress function value

df_nmds_stress = df_nmds$stress

df_nmds_stress

stressplot(df_nmds)

#Extract graphical data

df_points = as.data.frame(df_nmds$points)

df_points$samples = row.names(df_points)

names(df_points)[1:2] = c('NMDS1', 'NMDS2')

df_points

#Visualization

group = read.table("group.txt", sep='\t', header=T)

group = groupd2

colnames(group) = c("samples","group")

df = merge(df_points,group,by=group$samples)

df

mytheme = theme_bw() +

theme(axis.title.x = element_text(size = 18),

axis.title.y = element_text(size = 18),

axis.text.y = element_text(size = 12),

axis.text.x = element_text(size = 12),

panel.grid = element_blank(),

panel.border = element_rect(linewidth=1),

legend.position = 'none'

)

color=c('orange','yellow','turquoise2','steelblue1','purple')

p=ggplot(data=df,aes(x = NMDS1,y = NMDS2,fill = group)) +

mytheme + geom_point(aes(color = group), shape = 21, size=4) +

geom_hline(yintercept = 0, linetype = 2) +

geom_vline(xintercept = 0 ,linetype = 2) +

geom_text_repel(aes(label = samples),

size = 4) +

stat_ellipse(geom = "polygon",

level = 0.95,alpha = 0.2) +

scale_color_manual(values = color) +

scale_fill_manual(values = color) +

ggtitle(paste('Stress = ',round(df_nmds_stress, 3)))

p

#Figure 3 (b) ANOSIM

#Read data

library(vegan)

otu = read.delim('otu_table.txt', row.names = 1, sep = '\t', stringsAsFactors = FALSE, check.names = FALSE)

otu = data.frame(t(otu))

otu = df

group = read.delim('group.txt', sep = '\t', stringsAsFactors = FALSE)

#ANOSIM analysis (comparison between all groups, i.e. overall differences)

#Based on 999 permutations

#Bray Curtis distance

anosim_result = anosim(otu,group$group, distance = 'bray',permutations = 999)

dis1 = vegdist(otu, method = 'bray')

summary(anosim_result)

names(anosim_result)

anosim_result$signif #p value

anosim_result$statistic #R value

plot(anosim_result, col = c('red', 'orange','yellow','turquoise2','steelblue1','purple'))

1. **Figure 4.** The relative abundance of eukaryotic microbial communities at the level of phylum (a) and genus (b) in farmland and plantation soil.

#Figure 4 (a)

#Read data

phylum = read.delim('phylum.txt', row.names = 1, sep = '\t', stringsAsFactors = FALSE, check.names = FALSE)

# Calculate and rank the sum of abundance for each group

phylum$sum = rowSums(phylum)

phylum = phylum[order(phylum$sum, decreasing = TRUE), ]

#Selection of the top 10 taxa and merging of taxa outside the top 10 into ‘Others’

phylum_top10 = phylum[1:10, -ncol(phylum)]

phylum_top10['Others', ] = 1 - colSums(phylum_top10)

write.csv(phylum_top10, 'phylum_top10.csv', quote = FALSE)

png('barplot_plot.png', width = 1000, height = 700)

par(xpd = TRUE, mar = par()$mar + c(1, 3, 1, 16))

barplot(as.matrix(100*phylum_top9), col = c( 'orange', 'darkblue','tan3', 'dodgerblue', 'yellow', 'darkgreen', 'pink','mediumseagreen', 'deeppink2'), legend = rownames(phylum_top10), cex.axis = 2, cex.names = 2, ylim = c(0, 100), las = 1, width = 0.5, space = 0.5, beside = FALSE, args.legend = list(x = 'right', bty = 'n', inset = -0.18, cex = 2, y.intersp = 1.2, x.intersp = 0.7, text.width = 1)) Mtext (“Relative Abundance (%)”, cex = 2, side = 2, line = 4)

#Figure 4 (b)

#Read data

genus = read.delim('genus.txt', row.names = 1, sep = '\t', stringsAsFactors = FALSE, check.names = FALSE)

gensu$sum = rowSums(genus)

genus = genus[order(genus$sum, decreasing = TRUE), ]

genus_top14 = genus[1:10, -ncol(genus)]

genus_top14['Others', ] = 1 - colSums(genus_top14)

write.csv(genus_top14, 'genus_top14.csv', quote = FALSE)

barplot(as.matrix(100*genus_top14), col = c('darkgreen', 'yellow','lightblue','darkred','red', 'orange','aquamarine', 'darkblue', 'pink2', 'lightgreen','sandybrown', 'darkolivegreen3','purple','green4'), legend = rownames(phylum_top10), cex.axis = 2, cex.names = 2, ylim = c(0, 100), las = 1, width = 0.5, space = 0.5, beside = FALSE, args.legend = list(x = 'right', bty = 'n', inset = -0.18, cex = 2, y.intersp = 1.2, x.intersp = 0.7, text.width = 1)) mtext('Relative Abundance(%)', cex = 2, side = 2, line = 4)

1. **Figure 5. Spearman correlation heatmap about soil physiochemical factors with dominant phyla (a) and genera (b).**

#Figure 5(a)

#Read data

library(psych)

phylum = read.delim('phylum.txt', row.names = 1, sep = '\t', stringsAsFactors = FALSE, check.names = FALSE)

env = read.delim('env.txt', row.names = 1, sep = '\t', stringsAsFactors = FALSE, check.names = FALSE)

env = env[rownames(phylum), ]

#The p-value of the spearman correlation coefficient is corrected by default using the Benjamini & Hochberg method.

corr_matrix = corr.test(phylum, env, method = 'spearman', adjust = 'BH')

r = corr_matrix$r

p = corr_matrix$p

r = data.frame(r, ceck.names = FALSE)

r$phylum = rownames(r)

r = reshape2::melt(r, id = 'phylum')

r$phylum = factor(r$phylum, levels = rev(unique(r$phylum)))

names(r) = c('phylum', 'env', 'r')

head(r, 30)

# Heat mapping

library(ggplot2)

p1 = ggplot(r, aes(env, phylum)) +

geom_tile(aes(fill = r), color = 'gray') + scale_fill_gradientn(colors = colorRampPalette(c('cornflowerblue', 'white', 'red'))(21), limit = c(-1, 1)) +

theme(axis.text.x = element_text(angle = 90, hjust = 1, vjust = 0.5),

plot.title = element_text(hjust = 0.5)) +

scale_x_discrete(expand = c(0, 0)) +

scale_y_discrete(expand = c(0, 0)) +

labs(y = 'phylum', x = 'Environment', title = 'Spearman Correlation Plot', fill = 'rho')

p1

p = corr_matrix$p

r_sig = r

r_sig[p>0.05] = 0

r = data.frame(r, check.names = FALSE)

r_sig = data.frame(r_sig, check.names = FALSE)

#Figure 5(b)

#Read data

genus = read.delim('genus.txt', row.names = 1, sep = '\t', stringsAsFactors = FALSE, check.names = FALSE)

env = read.delim('genus.txt', row.names = 1, sep = '\t', stringsAsFactors = FALSE, check.names = FALSE)

env = env[rownames(genus), ]

corr_matrix = corr.test(genus, env, method = 'spearman', adjust = 'BH')

r = corr_matrix$r

p = corr_matrix$p

r = data.frame(r, ceck.names = FALSE)

r$genus = rownames(r)

r = reshape2::melt(r, id = 'genus')

r$genus = factor(r$genus, levels = rev(unique(r$genus)))

names(r) = c('genus', 'env', 'r')

head(r, 30)

library(ggplot2)

p1 = ggplot(r, aes(env, genus)) +

geom_tile(aes(fill = r), color = 'gray') + scale_fill_gradientn(colors = colorRampPalette(c('cornflowerblue', 'white', 'red'))(21), limit = c(-1, 1)) +

theme(axis.text.x = element_text(angle = 90, hjust = 1, vjust = 0.5),

plot.title = element_text(hjust = 0.5)) +

scale_x_discrete(expand = c(0, 0)) +

scale_y_discrete(expand = c(0, 0)) +

labs(y = 'genus', x = 'Environment', title = 'Spearman Correlation Plot', fill = 'rho')

p1

p = corr_matrix$p

r_sig = r

r_sig[p>0.05] = 0

r = data.frame(r, check.names = FALSE)

r_sig = data.frame(r_sig, check.names = FALSE)

1. **Figure 5. RDA of dominant phyla (c) and genera (d) with soil physiochemical factors.**

#Figure 5(c)

library(vegan)

read.table("phylum.csv",sep = ",",row.names = 1,header = T)

read.table("Env.csv",sep = ",",row.names = 1,header = T)

RDA = rda(phylum,Env,scale=TRUE)

RDA

summary(RDA)

s.RDA = RDA$CCA$u

#Display sample information

s.RDA

e.RDA = RDA$CCA$v

#Display environmental factor information

e.RDA

env.RDA = RDA$CCA$biplot

env.RDA

RDA.perm = permutest(RDA,permu=999)

RDA.perm

RDA.env = envfit(RDA,roscaGLU,permu=999)

RDA.env

#Drawing

plot(RDA)

plot(e.RDA)

plot(s.RDA)

#Figure 5(d)

library(vegan)

read.table("genus.csv",sep = ",",row.names = 1,header = T)

read.table("Env.csv",sep = ",",row.names = 1,header = T)

RDA = rda(genus,Env,scale = TRUE)

RDA

summary(RDA)

s.RDA = RDA$CCA$u

#Display sample information

s.RDA

e.RDA = RDA$CCA$v

#Display environmental factor information

e.RDA

env.RDA = RDA$CCA$biplot

env.RDA

RDA.perm = permutest(RDA,permu = 999)

RDA.perm

RDA.env = envfit(RDA,roscaGLU,permu = 999)

RDA.env

#Drawing

plot(RDA)

plot(e.RDA)

plot(s.RDA)

1. **Figure 6. Mantel test (a) of soil eukaryotic microbial communities and physiochemical parameters.**

#Figure 6(a)

library(linkET)

library(dplyr)

library(ggplot2)

#Read data

Envi = read.table("Env.csv",header = TRUE,row.names = 1,sep = ",")

envi

Tax= read.table("Tax.csv",header = TRUE,row.names = 1,sep = ",")

Tax

mantel = mantel_test(envi,Tax) %>%

mutate(rd = cut(r, breaks = c(-Inf, 0.2, 0.4, Inf),

labels = c("< 0.2", "0.2 - 0.4", ">= 0.4")),

pd = cut(p, breaks = c(-Inf, 0.01, 0.05, Inf),

labels = c("< 0.01", "0.01 - 0.05", ">= 0.05")))

mantel

set_corrplot_style(colours = c('cornflowerblue', 'white', 'red'))

qcorrplot(correlate(gene), type = "upper", diag = FALSE) +

geom_square() +

geom_couple(aes(colour = pd, size = rd),

data = mantel,

curvature = nice_curvature()) +

scale_size_manual(values = c(0.3, 0.6, 1)) +

scale_colour_manual(values = c('tomato3', 'springgreen4', 'grey') ) +

guides(size = guide_legend(title = "Mantel's r",

override.aes = list(colour = "black"),

order = 2),

scale_fill_gradientn(colors = RColorBrewer::brewer.pal(11,"Paired")),

colour = guide_legend(title = "Mantel's p",

override.aes = list(size = 3),

order = 1),

fill = guide_colorbar(title = "paerson's r", order = 3))

#Figure 6(b)

library(vegan)

library(tidyverse)

#Read data

Tax = read.delim('Tax.txt', row.names = 1, sep = '\t', stringsAsFactors = FALSE, check.names = FALSE)

# Perform a Hellinger transformation before sorting multidimensional data

Tax_hel = decostand(Tax, method = 'hellinger')

env = read.delim('env_table.txt', row.names = 1, sep = '\t', stringsAsFactors = FALSE, check.names = FALSE)

# Split the environment variables into two groups

rda_vp = varpart(phylum_hel, env['T','NN','AP'], env[c('pH', 'WC', 'AHN', 'OM', 'TN','AN','AK')])

rda_vp

plot(rda_vp, digits = 2, Xnames = c('T','NN','AP'), bg = c('darkseagreen2', 'lightsalmon'))
